# Contributions from the ovarian follicular environment to oocyte function

**DOI:** 10.21451/1984-3143-AR2018-0082

**Published:** 2018-08-17

**Authors:** Maite del Collado, Gabriella Mamede Andrade, Flávio Vieira Meirelles, Juliano Coelho da Silveira, Felipe Perecin

**Affiliations:** 1 Faculty of Animal Sciences and Food Engineering, Department of Veterinary Medicine, University of São Paulo, Pirassununga, SP, Brazil.

**Keywords:** cell-to-cell communication, cumulus-oocyte interactions, extracellular vesicles

## Abstract

The magnitude of oocyte’s role for embryo development is categorical. This unique cell contains the machineries and cellular components necessary to remodel male and female chromatin, to sustain early development and to, ultimately, generate a complete and complex individual. However, to gain these competences before fertilization, the oocyte undergoes several morphological, cellular and molecular changes during its lifetime enclosed in the ovarian follicle. This review will briefly revisit how the oocyte orchestrate the follicular cells, and how molecules transit to the oocyte from the innermost (cumulus) and outermost (antrum and granulosa cells) layers surrounding the follicle-enclosed oocyte. Finally, we will discuss the interferences of *in vitro* culture conditions in the communication of the oocyte with its surrounding cells and the potential strategies to modulate these communication systems to increase oocyte competence.

## Introduction

In females, before birth, cohorts of primordial germ cells migrate to the genital ridge and establish contact with pre-granulosa. Meiosis is triggered and the primordial follicles are formed by primary oocytes surrounded by small squamous granulosa cells ( Fortune, 1994 ; Eppig, 2001 ; Edson *et al*., 2009 ). The initial steps of follicular development are gonadotropin-independent and is essentially driven by locally secreted factors ( Eppig, 2001 ). During the growth phase these granulosa cells change from squamous to cuboidal originating the primary follicle ( Fortune, 1994 ; Eppig, 2001 ; Edson *et al*., 2009 ). As the oocyte grows, the zona pellucida, composed by glycoproteins, is formed constituting a physical barrier separating the oocyte and the granulosa cells ( Clarke, 2018 ). As granulosa cells proliferate and generate multiple layers, the follicle is denominated secondary follicle ( Fortune, 1994 ; Eppig, 2001 ; Edson *et al*., 2009 ). When the antrum appears within the follicle, granulosa cells are divided into cumulus cells and granulosa or mural cells ( Fortune, 1994 ; Eppig, 2001 ; Edson *et al*., 2009 ). From this moment, the oocyte slows or stops its growth, even though the follicle continues to grow, under the influence of mainly follicle stimulating hormone (FSH) up to the pre-ovulatory stage ( Fair *et al*., 1995 ; Eppig, 2001 ; Clarke, 2018 ). A peak of luteinizing hormone (LH) leads to mature cumulus-oocyte complex (COC) ovulation ( Fortune, 1994 ).

During the oocyte and follicle development, intense communication between the oocyte and the components within the follicular environment occurs to ensure the acquisition of oocyte developmental capacity. Until the follicle reaches the antral or tertiary stage, the folliculogenesis is independent of gonadotrophins and morphological and functional changes are controlled by paracrine signals ( Gilchrist *et al*., 2008 ). The oocyte commands these processes, in part, by oocyte-secreted factors (OSF). The molecules from the transforming growth factor-beta (TGF-B) superfamily are some of the most important OSF and, among them, Growth/differentiation factor 9 (GDF9) and Bone morphogenetic protein 15 (BMP15) have critical role during oocyte and follicle growth ( Gilchrist *et al*., 2008 ). GDF9 and BMP15 expression are detectable in oocytes as soon as the primordial or primary follicle stages, depending on the species ( Eppig, 2001 ). Oocyte releases GDF9 that acts in granulosa/cumulus cells through SMAD2/3 signaling ( Kaivo-Oja *et al*., 2003 ; Kaivo-Oja *et al*., 2005 ), whereas BMP15 signals through SMAD1/5/8 ( Moore *et al*., 2003 ; Pulkki *et al*., 2011 ). Both molecules can form homodimers and heterodimers and their proportions and functions vary in different species.

The importance of OSF for oocyte development was clearly demonstrated in mice once the knockout of these factors lead to sterile animals or animal with reduced fertility ( Dong *et al*., 1996 ; Eppig, 2001 ; Yan *et al*., 2001 ). A plethora of roles for GDF9 and BMP15 during oocyte maturation and folliculogenesis has been described. To exemplify, these OSF participate in the regulation of the oocyte growth factor KitL, increasing its expression in cumulus cells during oocyte growth and decreasing its expression when the oocyte is fully developed ( Eppig, 2001 ; Gilchrist *et al*., 2008 ); also, stimulate cell proliferation and drive the differentiation of granulosa and cumulus cells during folliculogenesis. These evidences indicate that the oocyte modulate the surrounding cells in order to gain developmental competence.

**Figure 1:**
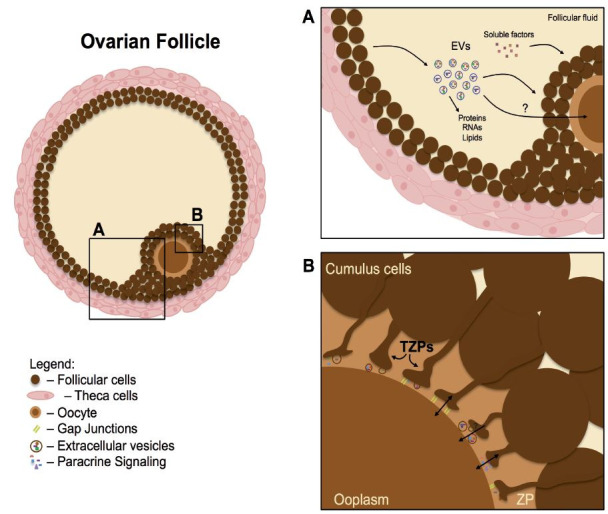
Communication within the ovarian follicle. Schematic representation of the intercellular communication within the antral ovarian follicle. **A.** Cellular communication in the follicular environment includes the secretion of paracrine factors (soluble factors) and a mechanism mediated by extracellular vesicles (EVs). These vesicles are present in follicular fluid and can carry and transfer macromolecules as mRNAs, microRNAs and proteins between granulosa cells, including the mural granulosa and cumulus. However, the eventually delivery to the oocyte of EVs’ contents transiting in the follicular fluid is yet to be experimentally observed **B.** The communication between the cumulus cells and the oocyte occurs by paracrine signaling. After the formation of the zona pellucida (ZP) this communication is also possible *via* transzonal projections (TZPs). At the bulk edges of TZPs gap junctions are established between cumulus cells’ membrane and the oolema. Structures like EVs have already been identified in the TZPs bulk edges, in the clefts formed between TZPs and the oolema.

However, the idea that the oocyte is the singular source of molecules that are accumulated during oocyte acquisition of competence has been contrasted by *in vitro* maturation studies, as reviewed by Robert and Gilbert (2018) . In cattle, the oocyte reaches its full-size when the follicle is around 3 mm in diameter ( Lodde *et al*., 2008 ). These follicles contain oocytes in germinal vesicle (GV), that can be classified as GV1, GV2 or GV3 according to their chromatin condensation stage, which is correlated to the transcriptional activity ( Luciano and Sirard, 2018 ). In GV1, the oocyte chromatin became partially condensed followed by a wide transcription decrease, while in GV3, the chromatin is totally condensed in a state of complete transcriptional inactivation ( Luciano *et al*., 2011 ; Lodde *et al*., 2013 ). Oocytes derived from follicles measuring 3 to 6 mm in diameter are usually used for *in vitro* production of bovine embryos, with development up to the blastocysts stage reaching 30 to 40%. When oocytes from larger follicles (around 8 mm) are used, blastocyst rate increase to 60% is observed ( Rizos *et al*., 2002b ). Even though oocytes either from 3-6 mm or 8 mm follicles are full-sized and transcriptionally inactive, the development competence is higher when they are kept longer within the follicular environment ( Lonergan *et al*., 1994 ), suggesting that cell-to-cell communications during the final stage of follicle maturation is decisive to increase the oocyte competence. Thus, follicular components and cumulus cells supply factors or molecules needed by the oocyte to reach its full competence. Here we will discuss the current knowledge about the communication within the ovarian follicle ( Figure 1 ), focusing on the movement of molecules from outward follicle components to the cumulus-oocyte complexes and from the cumulus cells to the oocyte.

## Relations between the follicular cells and the oocytes

Each follicular cell type within the follicular microenvironment play a role in supporting the oocyte growth, maturation and acquisition of competence. Indeed, several studies have associated follicular cells characteristics, such as gene expression and signaling pathways activity, with oocyte competence. For example, the expression of the gene *estrogen receptor 1* (*ESR1)* in granulosa cells and of the gene *versican* (*VCAN)* in thecal cells was positively associated with oocyte competence ( Matoba *et al*., 2014 ). In cumulus cells, genes such as *hyaluronan synthase 2* (*HAS2), Gremlim 1* (*GREM1)* ( Cillo *et al*., 2007 ), *regulator of G-protein signaling 2* (*RGS2)* ( Feuerstein *et al*., 2012 ) and *tumor necrosis factor- alpha inducible gene 6* (*TNFAIP6a)* ( Matoba *et al*., 2014 ) were positively associated with oocyte competence while the genes such as *inducible nitric oxide synthase* (*iNOS)* and *heme oxygenase 1* (*HO-1)* were negatively associated with competence ( Bergandi *et al*., 2014 ).

Studies that investigated gene expression in follicular cells and the ability of the oocyte to support development commonly identify associations or indirect effects of the follicular cells on the oocytes, rather than direct transfer of molecules among then. In spite of this, the direct transfer of molecules from the follicular cells to the oocytes play an important role for oocytes’ competence acquisition. The cumulus and corona cells, in close contact with the oocytes, provide small molecules to the oocytes through TZPs (and ultimately by gap junctions; ( Sugiura and Eppig, 2005 ; Conti *et al*., 2012 ). However, communication mechanisms allowing the exchange of large molecules, such as mRNAs and fatty acids ( Macaulay *et al*., 2014 ; del Collado *et al*., 2017b ), have only recent being described. Large molecules’ traffic between outer layers (i.e. mural granulosa) and inner layer (i.e. cumulus-oocyte complexes) of the ovarian follicle were recently described with the identification of extracellular vesicles (EVs) transiting in the follicular fluid ( da Silveira *et al*., 2012 ; Hung *et al*., 2015 ). Interestingly, the traffic of large molecules from cumulus to the oocytes has also been recently described through TZPs and ultimately by the transport mediated by EVs released into the cleft between the bulk end of the TZP and the oolema ( Macaulay *et al*., 2014 ; Macaulay *et al*., 2016 ).

## From the outer layers to the oocyte

### Extracellular vesicles

Extracellular vesicles are cell-secreted vesicles that can carry biomolecules such as proteins, mRNAs, miRNAs, metabolites and lipids ( Choi *et al*., 2013 ; Raposo and Stoorvogel, 2013 ). Extracellular vesicles are present in different body fluids including follicular fluid of diverse species such as equine, bovine and human ( da Silveira *et al*., 2012 ; Sang *et al*., 2013 ; Sohel *et al*., 2013 ; Santonocito *et al*., 2014 ; Navakanitworakul *et al*., 2016 ). These vesicles can be internalized by granulosa cells *in vitro* and *in vivo* ( da Silveira *et al*., 2012 ), and by cumulus cells *in vitro* ( Hung *et al*., 2015 ; da Silveira *et al*., 2017 ), suggesting their importance in intrafollicular communication. Additionally, extracellular vesicles are capable to facilitate the transfer mechanism of different macromolecules ( Di Pietro, 2016 ) not just between somatic cells but also between cumulus cells and the oocyte ( Macaulay *et al*., 2014 ; Macaulay *et al*., 2016 ), as discussed ahead. As the follicle develops extracellular vesicles quantity and content in the follicular fluid vary ( Navakanitworakul *et al*., 2016 ) and have different effect on cumulus expansion and oocyte gene expression ( Hung *et al*., 2015 ).

Recently, the role of extracellular vesicles was evaluated during cumulus-oocyte-complex *in vitro* maturation. Based on two independent studies using bovine as model the functional role of extracellular vesicles appears to be associated with EVs isolated from small follicles compared to large follicles. In the first study treatment of COCs with EVs from small follicles (3-5 mm) was capable to induce cumulus expansion and genes related to its function ( Hung *et al*., 2015 ). In a second study, extracellular vesicles isolated from 3-6 mm follicles were supplemented during COC *in vitro* maturation and early embryo development and were capable to induce changes in gene expression as well as global methylation and hydroxymethylation levels ( da Silveira *et al*., 2017 ). Among the genes influenced by extracellular vesicles treatment were DNMT3A (DNA methyltransferase 3 alpha), ACSL6 ((Acyl-CoA Synthetase Long Chain Family Member 6), CDH1 (Cadherin-1), REST (RE1-Silencing Transcription factor) and FADS2 (Fatty Acid Desaturase 2), which were increased in blastocysts following small EVs supplementation, during IVM and IVC, in comparison control treated COCs ( da Silveira *et al*., 2017 ). In addition to that was observed an increase in global methylation and hydroxymethylation upon small EVs supplementation during IVM and IVC in comparison with control treated COCs ( da Silveira *et al*., 2017 ). Additionally, da Silveira *et al*., (2017) observed the presence of PKH67 labelled EVs within TZPs suggesting the transfer of follicular fluid EVs to the oocyte; however, it still need to be confirmed ( da Silveira *et al*., 2017 ). These results indicate that the EVs present in the follicular fluid exert functions in cumulus-oocyte complexes probably by the direct transfer of EV cargo to COCs. The specific component of the EVs driving these effect is still unknown. The most well studied EV content, candidate to directly act into the COCs, are the microRNAs, which have been extensively studied in the follicular microenvironment in association with the oocyte competence.

### MiRNAs

MicroRNAs are small non-coding RNAs regulating gene expression that are present in cells or in follicular fluid and can be associated or not with EVs. MiRNAs can participate in the communication process between follicular cells and the oocyte ( Assou *et al*., 2013 ); thus, presenting different levels during follicular development ( McBride *et al*., 2012 ) as well as different predicted pathways involved in important cellular processes. For example, miR-130b is involved in increasing granulosa and cumulus cells proliferation and in oocyte maturation ( Sinha *et al*., 2017 ), probably by targeting SMAD5 and MSK1 (Mitogen- and Stress-Activated Protein Kinase 1) genes.

In order to investigate the potential roles of EVs from follicular fluid on the competence of bovine oocytes, our group conducted a series of experiments. We demonstrated the presence of different miRNAs cargos in EVs according to its origin – mural granulosa and COCs ( da Silveira *et al*., 2015 ; Andrade *et al*., 2017a ). Moreover, we demonstrated that the miRNAs transiting in EVs in the follicular fluid modulate signaling pathways, such as the PI3K-Akt pathway. Additionally, we show that higher or lower activity of the PI3K-Akt signaling pathway into follicular cells correlate with higher or lower developmental competence of the oocytes ( Andrade *et al*., 2017b ). Although a direct transfer of molecules from the follicular fluid-EVs to the oocyte has never been show, indirect influence of EVs present in the follicular fluid on oocyte competence was demonstrated using these and other approaches ( da Silveira *et al*., 2017 ).

## The inner layer: from cumulus cells to the oocyte

Denuded oocytes are not able to reach full competence to support development of an embryo, whereas denuded oocytes co-cultured with cumulus cells have result in increased blastocysts development rates ( Fukui and Sakuma, 1980 ; Zhang *et al*., 1995 ; Luciano *et al*., 2005 ). This demonstrates the importance of cumulus cells for the oocyte to acquire competence to support embryo development. The COCs are dynamic structures, and the communication among these cell types is intense. The unique architecture of the COC, in which the germ and soma cells are physically separated by a barrier, the zona pellucida, permits the communication mediated by paracrine signals as briefly discussed previously. However, the zona pellucida interposed between the oocyte and cumulus cell do not complete separate the germ and soma compartments, as transzonal projection (TZPs) extends from cumulus cell towards the oocyte. The TZPs allow the traffic of molecules from the cumulus cell to the oocyte by gap junctions or by extracellular vesicles ( Sutton *et al*., 2003b ; Macaulay *et al*., 2014 ).

### The transzonal projections (TZPs) and the traffic of small and large molecules to the oocyte

Besides paracrine signal, cumulus cells and oocytes crosstalk can occur by cumulus cells cytoplasmic projections through zona pellucida establishing a “bridge” connecting the cumulus cells and oocyte oolema, denominated transzonal projections (TZPs) ( de Loos *et al*., 1989 ; de Loos *et al*., 1991 ). In primordial follicles, oocyte and granulosa cells are in direct contact, however, during oocyte growth, the zona pellucida is formed and granulosa cells and oocyte become physically separated. The traffic of molecules between them will be dependent on the TZPs (reviewed by Clarke, 2017 ). In bovine, it was estimated that TZPs are functional until 9 hours of IVM; after 9 hours of IVM they begin to disrupt and after 18 hours of IVM there are no more active TZPs ( Macaulay *et al*., 2014 ; del Collado *et al*., 2017b ). The TZPs are composed by cytoplasmic filaments and two types of them have been characterized: the tubulin-TZPs and actin-TZPs ( Li and Albertini, 2013 ). Li and Albertini (2013) proposed two different functions depending on their structure: tubulin-TZPs are involved in cell adhesion function, while actin-TZPs, most abundant in COCs, are involved in cell communication. There are evidences of how the TZPs are formed and to what kind of regulation they are subjected. As reviewed by ( Clarke, 2018 ), there are two possible synthesis models: “stretching” and “pushing” models. The first proposes that, when zona pellucida appears, the granulosa cell bodies are stretched away from the oocyte and remaining attached to the original contact point; this model implicates that newborn granulosa cells are not able to synthesize TZPs. According to the “pushing” model, TZPs are generated by granulosa cells that grows toward the oocyte, and de novo TZPs synthesis could occur in proliferating granulosa cells. Although it was thought that only corona radiate cells could connect with the oolema by TZPs, recent studies have demonstrated that more distal layers also have these structures ( Jaffe and Egbert, 2017 ; El-Hayek *et al*., 2018 ). In bovines, it was estimated that around 3 thousand TZPs are present per oocyte ( Macaulay *et al*., 2016 ) and it was observed that mice cumulus cells can synthetized more than one TZP per cell ( El-Hayek *et al*., 2018 ), pointing an important communication route within the COC.

Molecules such as FSH and GDF9 have been shown to regulate the appearance of these projections. The study performed in mice showed that, in the absence of FSH, an unusual high number of TZPs appear in the oocyte surface, while FSH treatment induces TZPs retraction along with the acquisition of meiotic competence ( Combelles *et al*., 2004 ). The lack of GDF9 in mice oocytes lead to morphologically abnormal TZPs ( Dong *et al*., 1996 ; Carabatsos *et al*., 1998 ). Moreover, recently, an important work described how GDF9 could play an important role in TZP synthesis regulation *via* SMAD signaling and *Daam1* , *Fscn1* and *Myo10* proteins and possibly by another OSF, contributing to the regulation of this mechanism in mice ( El-Hayek *et al*., 2018 ). Although the description of TZPs occurred decades ago, we still do not fully understand what kind and extend of communication these projections permit between the oocyte and surrounding cells. At the end of each TZP, communications occur by gap-junctional traffic or, as recently proposed, mediated by extracellular vesicles ( Macaulay *et al*., 2014 ).

### Gap junctions

The gap junctions are transmembrane protein structures composed by six connexins. In COCs there are two kinds of gap junctions composed by two different types of connexins: the ones linking granulosa/cumulus cells and the ones between cumulus cells and the oocyte (at the bulk end of TZPs). Although this varies depending on the species, it seems that the most common pattern is that connexins 43 are more present in gap junctions between granulosa/cumulus cells, and the connexins 37 in gap junctions linking cumulus cells and the oocyte ( Nuttinck *et al*., 2000 ; Chang *et al*., 2016 ; Russell *et al*., 2016 ). Several studies in mice have demonstrated that both connexins are required to normal folliculogenesis and oogenesis ( Gershon *et al*., 2008 ; Chang *et al*., 2016 ). The connexins 43 are regulated by gonadotrophins and steroid hormones, and was recently demonstrated that they are negatively regulated by BMP15 ( Petrocelli and Lye, 1993 ; Granot and Dekel, 2002 ; Chang *et al*., 2014 ; Chang *et al*., 2016 ). Studies demonstrated that estradiol and FSH increase, while progesterone and LH decrease connexins 43 levels in many species such as human ( Petrocelli and Lye, 1993 ; Granot and Dekel, 2002 ). These communications are active from very early folliculogenesis and decrease gradually during growth of mid-antral follicles (2-6 mm) depending on the oocyte GV stage ( Lodde *et al*., 2007 ). During IVM in bovine, it was described that the number of gap junctions drop at 6-8 hours of culture, with the possibility of extending these communications using molecules that maintain the meiotic arrest in oocytes ( Lodde *et al*., 2013 ). Gap junction channels transport small molecules (< 1 kDa) such as ions, amino acids and metabolites important to oocyte development ( Thomas *et al*., 2004a ).

One of most important role of the gap junctions is the maintenance of meiotic arrest in oocyte. In mammals, oocytes are arrest in GV at diplotene stage of Profase I of meiose until preovulatory LH surge. It is well stablished that high level of cyclic adenosine monophosphate (cAMP) within oocyte maintain meiotic arrest, whereas a decreased of cAMP by phosphodiesterase (PDE3A) after LH peak leads to a meiotic resumption ( Sirard *et al*., 1998 ). Before the surge of LH, PDE3A activity in oocyte is inhibited by cGMP originated from cumulus cells via gap junctions inhibiting meiotic resumption ( Norris *et al*., 2009 ; Vaccari *et al*., 2009 ).

On the other hand, gap junctional communication allows a control of oocyte microenvironment by the traffic of molecules. The oocyte manages the intracellular pH against acidosis by gap junctions transport of pH regulators from cumulus cells until the oocyte is able to regulate pH by itself ( FitzHarris *et al*., 2007 ). In addition, the oocyte is metabolically restrictive during foliculogenesis, since it contains immature mitochondria not able to perform glycolysis ( Sutton-McDowall *et al*., 2010 ). Therefore, cumulus cells provide oocyte with metabolites such as pyruvate, that will be metabolized within the oocytes mitochondria during tricarboxylic acid cycle followed by oxidative phosphorylation to generate ATP ( Biggers *et al*., 1967 ; Sutton-McDowall *et al*., 2010 ). Moreover, cysteine and NAD(P)H traffic from cumulus cells to the oocyte via gap junction are involved in GSH (glutathione) synthesis, the most common antioxidant in the oocyte ( Sutton *et al*., 2003a ). Finally, the oocytes secrete paracrine factors to promote cumulus cell uptake *via* gap junctions of amino acids, such as alanine, histidine and leucine, that oocytes themselves transport poorly ( Eppig *et al*., 2005 ).

### Extracellular vesicles

There are increasing evidences that large molecules such as mRNA, miRNA and proteins could be transported from cumulus cells to the oocyte. Studies conducted by Claude Robert’s group ( Macaulay *et al*., 2014 ; Macaulay *et al*., 2016 ) demonstrated the RNA traffic from cumulus cells to the oocyte via TZPs. These studies described several important transcripts that cumulus cells provide to the oocyte at final stage of oocyte maturation, showing how these transcripts could traffic from the cumulus to the oocyte. Moreover, they showed the occurrence of extracellular vesicles (EVs) within a structure that resembles a synapse at the end of TZPs, which suggest an involvement of these vesicles mediating the transport. Recently, our group showed evidences of the transport of Fatty Acid Binding Protein 3 (FABP3) protein from cumulus cells to the oocyte *via* TZPs and an apparent increase of this traffic during the first 9 hours of IVM in bovines ( del Collado *et al*., 2017a ). Additionally, we have demonstrated the increase in lipids similar to the increase and accumulation of FABP3 protein, suggesting that this protein is involved in carrying lipids to the oocyte. In order to demonstrate the mechanism, we impaired actin filament polymerization thus blocking TZPs formation as well as FABP3 movement from cumulus to oocytes. Such strategy resulted in lower amounts of lipids accumulated within the oocyte. Based on these findings we are currently investigating the relationship between FABP3 and extracellular vesicles within the cumulus-oocyte-complex. In an attempt to demonstrate the possible transfer of extracellular vesicles from outside of cumulus cells to the oocyte we labeled EVs with PKH67 (membrane lipid dye) and verify the presence of labeled EVs within the zona pellucida, suggesting that these vesicles could use cumulus cells as bridges to achieve the ooplasm ( da Silveira *et al*., 2017 ).

## Disturbances and modulation of the communication within the ovarian follicle

Interactions inside the follicle microenvironment are coordinated and can lead to decreased oocyte competence if disturbed, or can potentially lead to increased reproductive success if adequately modulated. Understanding how follicular cells and the follicular fluid contents influence the oocyte competence acquisition is fundamental to develop strategies to protect the oocyte from deleterious conditions and to create protocols to improve assisted reproductive technologies. Since a fine coordinated crosstalk within COC is critic to oocyte competence, any exogenous particle or condition that modify cumulus cells or oocyte cellular or molecular characteristics could lead to an erroneous communication pattern, affecting oocyte competence. The most studied altered environment to which the oocytes are exposed is the *in vitro* culture condition. When immature oocytes undergo IVM several modifications appear (metabolic disturbances, epigenetic disturbances, and others) leading to a lower blastocyst rate, when compared to *in vitro* production (IVP) of *in vivo* maturated oocyte ( Rizos *et al*., 2002b ). This suggest that disturbances during oocyte maturation, corresponding to the final step of communication between the oocyte and the follicular cell, greatly impact the outcome oocyte competence acquisition.

*In vitro* maturation alters transcript profiles in cumulus cells and oocytes in several species ( Jones *et al*., 2008 ; Kues *et al*., 2008 ; Katz-Jaffe *et al*., 2009 ; Heinzmann *et al*., 2011 ; Yuan *et al*., 2011 ; Jiang *et al*., 2014 ). Since we already know the existence of RNA traffic from cumulus to the oocyte ( Macaulay *et al*., 2014 ; Macaulay *et al*., 2016 ), this traffic and, consequently, the oocyte quality, could be affected by IVM. However, evidences of differential RNA traffic within the COC under *in vivo* and *in vitro* conditions are yet to be demonstrated.

The excessive cytoplasmic lipid droplet accumulation that occur during the *in vitro* culture of gametes and embryos is extensively studied, and detrimental effects on competence are largely known ( Rizos *et al*., 2002a ; Seidel, 2006 ). We currently know that lipid accumulation occurs during IVM in both oocyte and cumulus cells and that it does not occur during *in vivo* maturation ( del Collado *et al*., 2016 ; del Collado *et al*., 2017a ; del Collado *et al*., 2017b ). Notably, we identified that functional modifications including lipid accumulation in *in vitro*-matured oocytes were not associated with coordinate transcriptional changes; rather, we observed marked alterations in expression patterns in the surrounding cumulus cells, that include a massive deregulation of transcripts related to fatty acid synthesis, accumulation, elongation and desaturation, β-oxidation and glycolysis ( del Collado *et al*., 2017a ). These data suggest that IVM drastically interferes with the cumulus cells transcription, while modifications in lipid quantity occurs in both cumulus cells and oocyte. Considering the intense communication within COC, we could suggest that these alterations could modify the synthesis of fatty acids into cumulus cells and the quantity of molecules trafficking between soma and germ cells. This principle was given proof by the investigation of lipid traffic from cumulus cells to oocytes via a lipid-carrying protein (FABP3) and TZPs ( del Collado *et al*., 2017b ). We identified FABP3 within TZPs in bovine immature and *in vitro* mature oocytes. Moreover, we demonstrated that FABP3 protein and lipids increased in *in vitro* matured cumulus cells and oocytes, but not in *in vivo* counterparts, suggesting that in *in vitro* conditions the increase of lipid synthesis and accumulation in cumulus cells is, at least partially, transferred to the oocytes via TZPs.

The glucose metabolism is also affected by IVM. COCs undergoing IVM produce higher levels of lactate ( Khurana and Niemann, 2000 ) and cumulus cells after IVM have higher expression of glycolysis related genes when compared to *in vivo* maturation ( del Collado *et al*., 2017a ). If *in vitro* maturated cumulus cells metabolized more glucose and produced higher lactate, it could be implied that these cells provide to the oocyte higher level metabolites from glycolysis by gap junctions. Herein, we expose evidence suggesting that IVM affect COC communication by several different ways, being able to influence the oocyte competence and embryo development. We pointed out some mechanisms and pathways affected by *in vitro* maturation and their implications in oocyte competence. However, some important questions remain: can we modulate COC communication during IVM? Is this strategy viable to increase oocyte quality?

Increase communication time between cumulus cells and the oocyte is a strategy under investigation in recent years by the use of pre-maturation systems before IVM. The pre-maturation are proposed to maintain TZPs and gap junctions functional for longer periods during *in vitro* culture. This is achieved with the use of substances that modulate cAMP and the meiotic resumption ( Gilchrist and Thompson, 2007 ). Studies have demonstrated that pre-maturation systems increase oocyte quality and blastocysts rates during IVP in several mammals ( Thomas *et al*., 2004b ; Gilchrist and Thompson, 2007 ; Li *et al*., 2016 ; Sugimura *et al*., 2018 ). At a glance, the strategy to improve oocyte quality by increasing cumulus cells-oocyte communication during IVM could provide to the oocyte similar amounts of molecules, such as mRNA and metabolites, as *in vivo* maturated oocytes. However, one thing that has to be taken into account is that, during IVM, oocytes’ population is very heterogeneous containing oocytes at different GV stages. These differential GVs stages could be differently affected by the pre-maturation systems. Oocytes in GV1 could benefit from this system, accumulating more molecules from cumulus cells, whereas more advance GV3 oocytes, with fewer gap junctions, could be adversely affected by *in vitro* maturation without any advantage by pre-maturation. Moreover, we have to consider that, as previously described, *in vitro* maturated cumulus cells are negatively affect by *in vitro* culture, so the quantity and type of molecules transiting from cumulus to the oocyte could be non-physiological and not beneficial as expected.

On the other hand, a different strategy to modify COC communication is the supplementation with EVs during IVM. There are studies demonstrating a positive influence of supplementation with extracellular vesicles during IVM in cumulus cells expansion, blastocyst rate, blastocysts cryotolerance and gene expression in the COCs ( Hung *et al*., 2015 ; Lopera-Vasquez *et al*., 2016 ). Furthermore, supplementation with EVs from follicular fluid of 3-6 mm follicles during IVM and IVC altered the expression of metabolic and developmental related genes, miRNA profile and epigenetic marks in blastocyst ( da Silveira *et al*., 2017 ). These studies suggest that EVs play roles during oocyte maturation and embryo development. However, to develop such strategy, is important to understand the role of these extracellular vesicles within the ovarian follicle as well as the specific roles of their contents in order to supplement the right ‘messages’ to cumulus-oocytes-complexes during maturation.
